# Older adults can outperform younger adults in creative problem solving

**DOI:** 10.1186/s12877-025-06707-w

**Published:** 2026-01-20

**Authors:** Barbara Ozkalp-Poincloux, Mathieu Cassotti, Émilie Salvia, Gaelle E. Doucet, Anaëlle Camarda

**Affiliations:** 1 Université Paris Cité, LaPsyDÉ, CNRS, 46 rue Saint Jacques, Paris, F-75005 France; 2 Institute for Human Neuroscience, Boys Town National Research Hospital, Boys Town, NE USA; 3 Department of Pharmacology and Neuroscience, Creighton University, Omaha, NE USA; 4 Nantes Université, Laboratoire de Psychologie des Pays de la Loire (LPPL UR 4638), Nantes, F- 44000 France; 5 Institut Supérieur Maria Montessori (ISMM), Paris, France

## Abstract

**Background:**

This study investigated the impact of aging on creative problem solving, focusing on the ability to resist fixation and generate original ideas. Previous research has suggested that creativity peaks in middle adulthood, followed by a decline that is linked to reduced executive functions. However, emerging evidence challenges this view, highlighting that older adults may utilize broader associative networks and prior knowledge to maintain their creativity.

**Method:**

Young adults, middle-aged adults, and older adults completed a creative task requiring them to generate solutions to a problem while resisting fixation. The participants also rated the creativity of their ideas, and their executive functioning was assessed.

**Results:**

The results revealed that older adults generated more original and expansive responses than younger and middle-aged adults did, particularly during initial responses. This advantage appears to be linked to leveraging distant associations rather than superior executive control. Temporal analysis revealed that older adults performed better in generating original ideas early in the task but did not sustain this advantage as the task progressed.

**Conclusion:**

These findings suggest that while aging is associated with some types of cognitive decline, it also fosters unique cognitive strengths that can enhance certain aspects of creativity. The findings of the current study underscore the need to reconsider stereotypes about aging and creativity and highlights the interplay of executive and associative processes in creative ideation.

## Introduction

When most people imagine creative workers gathering around Post-it notes and throwing out great ideas that will allow them to engage in innovation for their industries, they are unlikely to imagine a group of people over 60 years of age. They think more of a group of young hipsters because there is a certain stereotype that remains regarding the development of creativity in adults and the effects of aging on cognition [1].

Early investigations in psychology provided support for the hypothesis of a decline in creativity in late adulthood by showing that divergent thinking performance (i.e., the ability to generate many creative ideas) increases during adulthood, peaks at approximately age 40 and then decreases [2–4]. Both cross-sectional and longitudinal studies of divergent thinking across the lifespan [2, 5] have reported curvilinear trends, with a progressive decline after 40 years of age in terms of the quantity and creative quality of the idea generated by the participants.

Interestingly, the results of these studies are consistent with those showing a decline with age in high-level cognitive processes that are known to be involved in creativity, such as inhibitory control or executive functions in general [1, 6]. Indeed, the cognitive approach to creativity has shown that the ability to generate creative ideas involves the inhibition of cognitive biases known as fixation effects [7]. Thus, when individuals seek to explore original solutions to a problem, they tend to become stuck in a limited number of classic ideas that are easily accessible in memory [8, 9]. For example, when individuals are asked to design solutions to prevent an egg from breaking when dropping it from a height of 32 feet, typical solutions from similar contexts quickly come to mind [8–11]. Most adult responses involve the use of a passive device to mitigate the impact, protect the egg, or slow its descent. Participants in such an experiment tend to fixate on these conventional solutions, often overlooking more original approaches, such as using a living device or altering the egg’s natural properties (e.g., training a bird to catch the egg mid-fall or freezing the egg before the drop). This phenomenon, called the “fixation effect”, has been widely documented in the literature as a major obstacle to creative idea generation [7]. In this context, recent studies have reported that overcoming these fixation effects requires the inhibitory control of intuitive solution strategies that are not relevant to solve the problem creatively. Camarda et al. [12] used a dual-task paradigm to reduce participants’ inhibitory control resources while performing a creative task and reported that the inhibitory control load decreased creative ideation in terms of both fluidity and originality. These findings align with the triadic model of creativity [7]. Drawing inspiration from models developed in the fields of reasoning and decision-making [13] this model posits that the ability to explore original ideas to solve a problem relies on the interaction of three distinct systems, namely, intuitive, cognitive control and deliberative systems. The intuitive system, which leads to fixation, generates initial responses to the problem, whereas the cognitive control system inhibits these responses, thereby allowing the deliberative system to explore more original solutions. From a developmental perspective, the triadic model posits that the ability to generate creative ideas relies on the competitive balance between closely associated intuitive responses that lead to fixation and cognitive control processes. Consequently, both the strength of intuitive system activation and cognitive control capacities may vary with age and help explain the nonlinear developmental trajectories observed during childhood and adolescence [9, 10]. While cognitive control tends to develop in a linear fashion, certain fixation effects also emerge during childhood or expertise. Thus, some younger children or novices may [14] exhibit better problem-solving performance not because they are better at overcoming fixation, but because the nature of their fixation differs from that of older children or adults. This model therefore suggests that understanding how individuals generate original solutions to a problem requires qualitatively considering both the nature of fixation and cognitive control capacities.

In line with this hypothesis emphasizing the key role of cognitive control, studies that use divergent thinking tasks (e.g., the Alternative Uses Task [AUT]) have consistently shown that both creativity and demand in executive control are greater for later responses than for initial responses [15, 16]. This serial order effect, which reflects how ideas that arrive later tend to be more creative than the earlier ones, has been widely observed across various tasks and samples [15]. While the classic explanation posits that time allows individuals to access more remote and less obvious associations of concepts within a semantic network, leading to unique and more creative responses, recent insights underscore the role of executive processes in shaping this effect [17–19]. Thus, the serial order effect in creative thinking may reflect top-down executive processes that unfold over time to overcome interference from more obvious ideas.

Creativity is thought to rely, in part, on the ability to inhibit fixation effects. Since inhibitory control follows an inverted U-shaped developmental trajectory—peaking around 30 to 40 years of age [6] —one might expect creative performance to follow a similar age-related pattern. Moreover, results regarding the serial order effect suggest that the decrease in creativity between young adults and older adults should be more pronounced for the last response, as the demand for executive resources is at its highest level at this stage. Nevertheless, recent findings seem to challenge the peak and decline hypothesis, suggesting that older people may be more creative than previously thought [1]. Indeed, some studies have failed to observe an age-related decline in creativity between young and older adults [15, 16, 20–22]. This lack of an age effect is particularly noticeable when older adults have more time to respond [21]. A recent study reported that creativity seems to remain relatively stable in older individuals, except for certain creative abilities that are influenced by abstract reasoning, which appear more vulnerable to aging [23, 24].

Additional support for this view comes from neuroimaging identifying a positive association between higher levels of creativity and increased functional connectivity (i.e., coupling) between the default and executive control networks, which is an effect that is more pronounced in older adults than in younger adults [16]. Additionally, greater creativity in older adults has been linked to enhanced whole-brain functional connectivity in the resting state [25]. At a cognitive level, these results suggest that older adults might be able to exploit a larger amount of prior knowledge and use more distant associations when generating multiple original ideas in divergent thinking tasks. This mechanism could compensate for the decline in executive resources and lead to no difference being found between young and older adults in regard to creativity tasks [15, 16]. Notably, some investigations have shown that older adults may perform better in creativity tasks when only originality is accounted for or in tasks that involve remote associations [1, 26, 27]. For example, Leon et al. [27] reported that older adults provide more unique responses than young adults do on two verbally based divergent thinking tasks when no time limit is imposed. The fact that the absence of time pressure leads to better performance in creative tasks in older adults raises questions about the nature of the cognitive processes that enable them to be more creative.

Recent advances in the triadic model of creativity [7, 14] suggest that while executive functions are necessary to overcome intuitive fixation effects, some individuals intuitively manage to generate original responses. When participants are asked to provide the first solution that comes to mind to a creative problem-solving task under time pressure—which is intended to minimize the activation of the executive system [28] —the vast majority provide responses within the fixation solution path [14]. However, a small subset of participants (fewer than 10%) succeed in generating creative ideas during the initial response stage, which may be more related to higher associative processes than to a greater level of executive resources [14]. These results confirm that, while intuitive thinking leads to fixation effects for the majority of individuals, some are able to overcome fixation intuitively — demonstrating that an intuitive form of creativity is also possible. These findings underscore the need to consider time constraints to better understand the processes involved in creative ideation and the impact of aging on it.

One of the limitations of the lifespan studies mentioned above is that they have focused almost exclusively on the development of divergent thinking using tasks that do not involve problem solving [1]. This is notably the case with the alternative uses task, in which participants propose alternative uses without a specific goal. Consequently, the age advantage that has been described in divergent thinking tasks could result from a better ability to connect distant knowledge without facilitating the ability to creatively solve problems that elicit fixation effects. However, the temporal dimension has been rarely considered in such studies. This is regrettable for at least two reasons. First, previous studies have shown that older people can match the performance of younger people when given sufficient time; second, the demand for executive resources increases with time and is greater for the last ideas generated than for the first. Therefore, having access to the temporal course of ideas, alongside executive measures, could provide insights into the processes involved in the effects of age on the ability to resist fixation in problem solving.

In this context, the aim of our study was to examine the impact of age on the ability to resist fixation effects and generate original solutions to a problem from young to older adulthood. To this end, we recruited a group of young adults, middle-aged adults, and older adults who completed a computerized version of the egg task [14], along with a questionnaire that aimed to measure their executive abilities. Drawing on tasks used in neuroimaging [19], this version of the egg task requires participants to press a button each time an idea comes to mind before being prompted to write it down. This approach allowed us to capture the temporal distribution of ideas. Additionally, we included an evaluation phase where participants rated the creativity of their own ideas on a 7-point Likert scale to obtain a metacognitive monitoring score (i.e., the ability to correctly evaluate the creativity of the generated solutions). Recent studies have suggested that metacognitive control is a fundamental component of creative ideation [29]. We hypothesized the following:

- H1: In line with the hypothesis of an age-related decline in executive functions and given that this creative task involves inhibitory processes, older adults should be less creative than middle-aged and younger adults. Furthermore, consistent with data on the serial order effect, this decline should be more pronounced toward the end of the task.
- H2: In contrast, if older adults simply require more time to activate executive processes, they should demonstrate comparable or even superior performance to the other age groups, but only in their later responses.
- H3: Finally, if the superiority of older adults in originality results from a greater ability to activate remote associations in memory, they may demonstrate greater creative performance from the very first ideas, which would be consistent with recent findings highlighting the existence of creative intuition [14].

## Method

### Participants

A total of 249 participants were recruited for the experiment, namely, 83 young adults (*M* = 20 years; *SD* = 1.58; *Min* = 18; *Max* = 23; 48.2% male), 86 middle-aged adults (*M* = 38.4 years; *SD* = 4.55; *Min* = 29; *Max* = 46; 46.5% male), and 80 older adults (*M* = 68.4 years; *SD* = 3.71; *Min* = 62; *Max* = 78; 50% male). All participants provided written consent and were tested in compliance with national and international guidelines governing the use of human research participants. The sample size was determined a priori using G*Power 3.1.9.2 [30], which indicated that a minimum of 102 participants would be needed to detect a small effect size of 0.20 (based on Cohen’s effect size conventions), with a power of 0.80 and an α of 0.05.

The first nine items and two example items from the Wechsler Adult Intelligence Scale (WAIS-IV) were presented to older adults. Older adults who scored more than two standard deviations below the mean were excluded from the analysis (*N* = 2). Additionally, participants who indicated that they did not understand the task instructions were removed from the analysis (middle-aged adults: *N* = 1; older adults: *N* = 1). The final sample consisted of 83 young adults (*M* = 20 years; *SD* = 1.58; *Min* = 18; *Max* = 23; 48.2% male), 85 middle-aged adults (*M* = 38.4 years; *SD* = 4.57; *Min* = 29; *Max* = 46; 45.9% male), and 77 older adults (*M* = 68.3 years; *SD* = 3.53; *Min* = 64; *Max* = 78; 52% male).

### Tasks

#### Creative solving problem task

All the participants were asked to perform a creativity task within 10 min. The task consisted of the generation of as many original solutions as possible to the following problem: “Ensure that a hen’s egg does not break when dropped from a height of 32 feet” [7–9, 11, 12, 31]. The participants were instructed that there were no right or wrong answers and that they had to provide as many creative solutions to the problem as possible in the allotted time. To do so, participants were instructed on the first screen to click on a “I have an idea” button as soon as a novel idea came to their mind. A second screen then appeared, on which they were asked to type their new solution and evaluate its level of creativity on a Likert scale ranging from 1 (not at all creative) to 7 (very creative). Once the idea was submitted, the first screen returned, allowing participants to develop another novel idea, and so on, until the time was up.

To assess creativity, we applied a well-established originality measure drawn from prior research on the egg task. This measure involved categorizing solutions into different groups [7–9, 11, 12, 31]. A trained evaluator assigned each participant’s response to one of 10 meta-categories, which represented broad solution types identified through the concepts–knowledge (C–K) design method [32]. A second trained rater independently coded 50% of the responses, selected across age groups, to assess reliability. Inter-rater agreement was excellent (91.57%). All categories and detailed information regarding the use of the egg task are available online on OSF (https://osf.io/j984z/files/osfstorage/66389a6c419d002727fea01a). On the basis of previous studies, solutions within the three most common meta-categories for adults (i.e., reducing shock, protecting the egg, and slowing the fall) were classified as fixation responses. Solutions that fell into one of the seven other meta-categories were considered expansion responses (i.e., creative ideas), such as using a living object or modifying the egg’s natural properties. Examples of solutions categorized as fixation or expansion within each of the ten meta-categories are presented in Table 1. Moreover, to facilitate comparisons between the results of the present study and previous findings using a single originality score, we calculated an “originality score” for each participant using the consensual assessment technique proposed by Amabile [33]. Two independent raters were instructed to evaluate each idea on a five-point rating scale ranging from 1 (not original at all) to 5 (highly original). The raters displayed a satisfactory intraclass correlation coefficient (0.89). Each participant was then scored on the mean rating of the originality scores of all of the solutions. In accordance with Benedek and Lebuda’s [29] study, we computed a measure of metacognitive control at the response level (MCC-R). This was calculated by subtracting the originality score (rated on a 5-point Likert scale) from participants’ judgments of the creativity level of their ideas (rated on a 7-point Likert scale). Both scales for evaluating creativity were normalized using a min-max calculation to enable proper comparison between the two scales. This measure reflected the participants’ ability to accurately judge their creativity. A positive value of the MCC-R indicated that participants tended to overestimate the level of their creativity, whereas a negative value indicated that they underestimated it.

**Table 1:** Examples of solutions that could be categorized as ideas belonging to meta-categories classified in fixation and meta-categories classifies as in expansion

Meta-category	Type	Example of solutions
Reducing the shock	Fixation	*Put a mattress on the floor.*
Protecting the egg		*Wrap the egg in a blanket.*
Slowing the fall		*Tie a parachute to the egg.*
Interrupting the fall	Expansion	*Catch the egg with a net*
Acting before the fall		*Drop the egg at a height of 11 m*
Using a living device		*Train an eagle to take down the egg*
Acting after the fall		*Replace the broken egg with an unbroken one*
Modifying the properties of the egg		*Freezing the egg*
Modifying/Act on the properties of the environment		*Throw the egg on the Moon.*
After the fall		*Dropping the egg in zero gravity*

In summary, a participant’s creativity was evaluated according to four classical indicators used in the egg task, namely, the overall fluency (i.e., the number of solutions given by a participant), the fixation score (i.e., the total number of solutions generated within the fixation meta-categories), the expansivity score (i.e., the total number of solutions generated in the expansive meta-categories), and the originality score (i.e., rated on a 5-point Likert scale, according to the CAT) Table 2.

**Table 2:** Descriptive results of participants based on age, level of education, typing speed, gender, and previous knowledge of the egg task (see the supplementary materials for a comprehensive analysis of group differences based on gender, educational level, prior knowledge of the egg task, and writing speed: https://osf.io/a4d9n/files/osfstorage/684ab719c2209fbf4b85b35a)

	Young Adults	Middle-aged adults	Older adults
Age			
*N*	83	85	77
*Range*	18–23	29–46	64–78
*Mean*	20	38,4	68,3
*Standard Deviation*	1,58	4,57	3,53
Niveau d’éducation			
*Range*	0–17	0–17	0–17
*Mean*	11,6	13,2	12,1
*Standard Deviation*	3,82	3,54	3,26
Typing speed (seconds)			
*Range*	3.07–25.3	6.22–40.8	8.66–71.3
*Mean*	11,3	14,7	22,2
*Standard Deviation*	4,35	6,26	10,1
% of Male	48,2	45,9	52
% Knowledge of the Egg Task	10,84	16,47	5,19

To examine the temporal dynamics of fixation and expansion, we divided the 10-minute idea-generation task into five 2-minute periods. Because fixation and expansion ideas were analyzed separately, shorter intervals (e.g., 1 min) resulted in too few responses per category—especially toward the end of the task—to allow reliable model estimation. This choice is also consistent with Beaty and Silvia’s data [17], which show that idea fluency decreases rapidly over time, dropping to fewer than one response per minute on average at the end of the task. Therefore, 2-minute intervals provided an appropriate balance between temporal resolution and sufficient data density for comparing fixation and expansion responses. Additionally, on the basis of the study by Camarda et al. [14], which showed that the very first response could be intuitively creative, we also determined whether this first response belonged to the fixation or expansion category.

### Executive functioning questionnaire

The participants were asked to complete the Adult Executive Functioning Inventory (ADEXI) [34], which is a 14-item questionnaire that aims to assess self-reported executive functioning abilities related to working memory and cognitive inhibition in daily life. Using a five-level agreement scale, participants had to indicate how much an item related to them (1 = definitely not true, 2 = not true, 3 = partially true, 4 = true to 5 = definitely true). The items in the working memory component included “I have difficulty remembering lengthy instructions”, “I sometimes have difficulty remembering what I am doing in the middle of an activity” and “I have difficulty coming up with a different way of solving a problem when I get stuck”. In the inhibitory control process, examples items are “I sometimes have difficulty stopping myself from doing something that I like even though someone tells me that it is not allowed”; “People who I meet sometimes seem to think that I am more lively/wilder than other people my age”; and “I have a tendency to do things without first thinking about what could happen”. For each component, we summed the evaluation of each statement to obtain a score for working memory and a score for inhibition. The Cronbach’s alpha for the full scale indicates high internal consistency (α = 0.86).

### Procedure

The experiment took place online on the *Qualtrics* platform. Participants first received general instructions about the study and provided their consent to participate. They were then asked to report their age and gender. To control for potential differences in knowledge or education, participants were asked about their level of education and whether they had previously heard of the egg task by answering a yes-or-no question. Participants indicated the highest diploma they had obtained, selecting from six major levels of education in France, ranging from no diploma to degrees (e.g., middle school diploma to master’s degree or higher). These diploma categories were subsequently converted into a numerical variable, with values ranging from 0 (no diploma) to 17 (master’s degree or higher).

A measure of typing speed was then proposed, in which participants were asked to type the sentence “Children are playing in the park with a ball” as quickly as possible so that we could assess intergenerational differences in typing on a keyboard. Afterward, the participants were presented with the creativity task and the ADEXI questionnaire. To fully understand the instructions, the participants engaged in quick training session (1 min) by answering the following question: “Cite as many colors as possible”. Moreover, the older participants were asked to solve the first items of the WAIS-IV, the purpose of which was to exclude those with cognitive decline outside the normal range. The experiment lasted approximately 20 min.

## Results

### Intercorrelations analysis

**Fig. 1:**
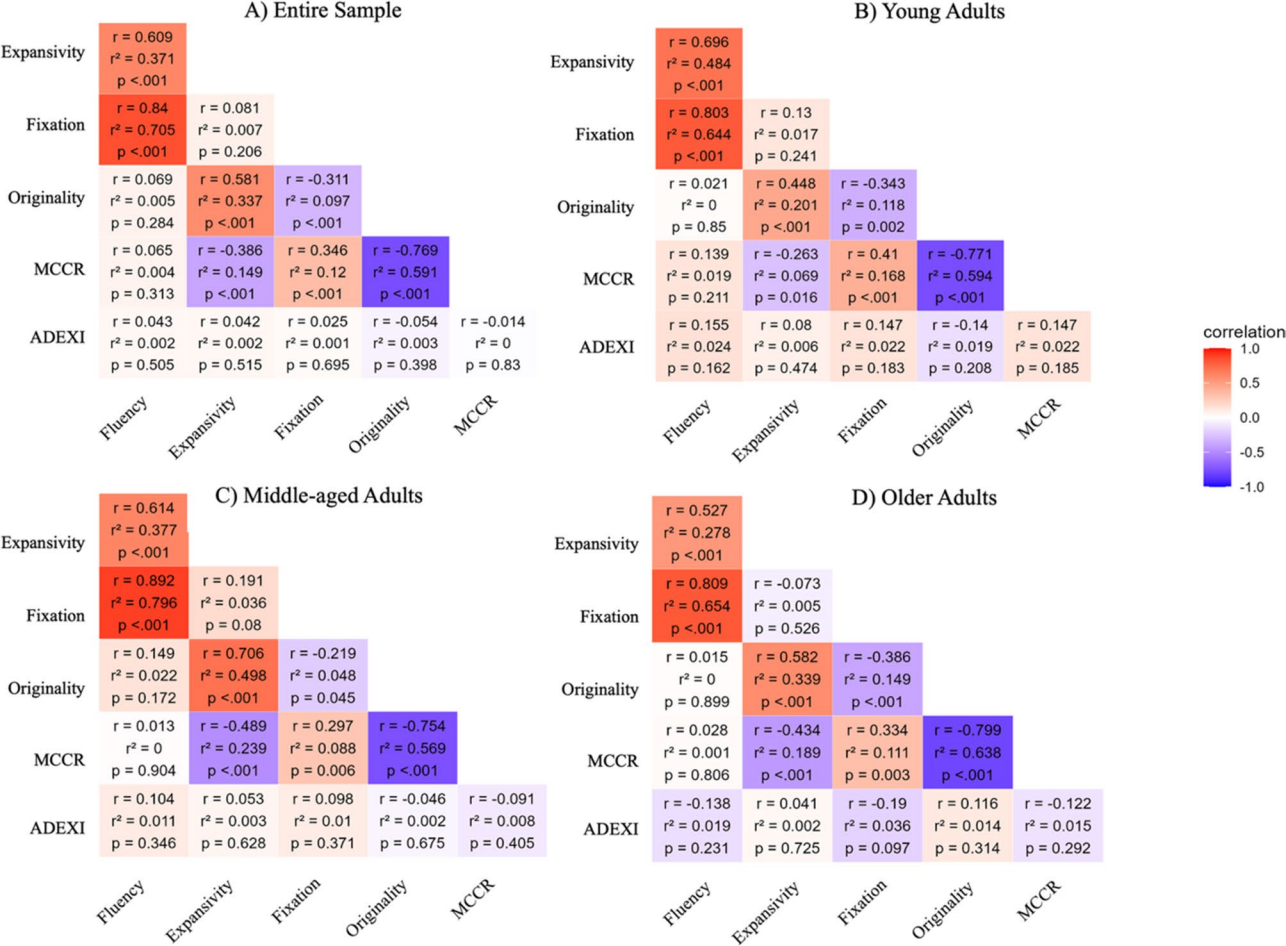
Intercorrelations between each creativity score for the entire sample (**A**) and across every age group: **B**) Young adults; **C**) Middle-aged adults and **D**) Older adults

For the entire sample, fluency was found to be positively correlated with both expansivity (*r* =.61, *p* <.001) and fixation (*r* =.84, *p* <.001; Fig. 1). However, the two variables were found to be not correlated (*r* =.08, *p* =.20). Furthermore, the originality score was found to be positively correlated with expansivity (*r* =.58, *p* <.001) but negatively correlated with the number of ideas in fixation (*r* = −.31, *p* <.001). With respect to the MCC-R score, we found that the more participants overestimated the creativity of their responses (i.e., as reflected by a positive MCC-R score), the more they tended to provide fixation responses (*r* =.35, *p* <.001) and the less they provided expansive and original responses (for expansivity: *r* = −.37, *p* =.002; for originality: *r* = −.77, *p* <.001). ADEXI scores did not correlate with creativity scores (all *p* values > 0.30). Within each age group, the same pattern of correlations was observed (see Fig. 1 for details).

### ADEXI

To examine the effects of age groups (young, middle-aged, and older adults) on ADEXI scores for working memory (WM) and inhibition (I), we conducted analyses of variance (ANOVAs) with age group serving as a between-subjects factor. Effect sizes were evaluated using partial eta squared (partial *η²*) or Cohen’s *d*, as appropriate. The ANOVAs indicated a significant main effect of age (WM: *F*(2, 242) = 6.99, *p* <.001, partial *η²* = 0.055; I: *F*(2, 242) = 4.60, *p* <.001, partial *η²* = 0.037). Younger adults reported higher self-assessed levels of working memory and inhibition (WM: *M* = 18.9, *SD* = 6.75; I: *M* = 14.5, *SD* = 4.82) than middle-aged (WM: *M* = 15.6, *SD* = 5.27; *t*(242) = 3.561, *p* <.001, *d* = 0.54; I: *M* = 13.1, *SD* = 4.3; *t*(242) = 2.166, *p* =.031, *d* = 0.31) and older adults did (WM: *M* = 16.3, *SD* = 5.87; *t*(242) = 2.75, *p* =.006, *d* = 0.41; I: *M* = 12.5, *SD* = 3.76; *t*(242) = 2.916, *p* =.004, *d* = 0.46). Importantly, no significant differences were found between middle-aged and older adults (WM: *p* =.465; I: *p* =.419).

### Creative problem-solving task

To test H1, H2 and H3, we examined the effects of age groups (young, middle, and older adults) on the number of proposed solutions (i.e., fluency) over time, we conducted a repeated-measures ANOVA with age group serving as a between-subjects factor and type of idea (fixation or expansion) and period (5 periods of two minutes) serving as within-subjects factors. Notably, the significant interactions reported below were still significant after controlling for the ADEXI scores, typing speed, and also when participants indicating their knowledge of the egg task were excluded (see supplementary data 2).

For the fluency measure, the ANOVA revealed a main effect of type of idea (*F*(1, 242) = 139.49, *p* <.001, partial *η²* = 0.37) and time (*F*(4, 242) = 410.23, *p* <.001, partial *η²* = 0.63); however, it did not reveal a significant main effect of age group (*F* < 1, *p* =.42). A significant interaction effect between the type of idea and age was observed, *F*(2, 242) = 4.14, *p* =.017, partial *η²* =0.033. Planned comparison revealed that more ideas were generated related to fixation (*M* = 5.77, *SD* = 3.37) than to expansion (*M* = 2.82, *SD* = 2.31). However, the older adults generated significantly fewer ideas related to fixation (*M* = 5.32, *SD* = 3.13) than did the middle-aged adults (*M* = 6.38, *SD* = 3.84, *t*(242) = 1.99, *p* =.047, *d* = 0.30). Critically, older adults generated more ideas related to expansion (*M* = 3.31, *SD* = 2.17) than younger adults did (*M* = 2.57, *SD* = 2.49, *t*(242) = 2.06, *p* =.041, *d* = 0.32) and tended to generate more ideas related to expansion than middle-aged adults did (*M* = 2.61, *SD* = 2.20, *t*(242) = 1.94, *p* =.053, *d* = 0.32).

Critically, the results indicated a significant three-way interaction between age group, time and type of idea, *F*(8, 242) = 2.36, *p* =.016, *η²p* =.02.

**Fig. 2:**
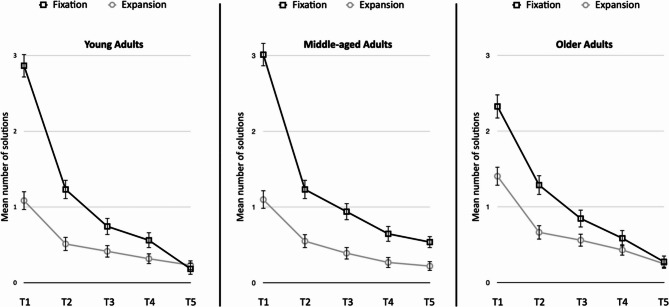
The mean number of responses in the fixation and expansion categories as a function of the 2-minute time period and age group

The results of a planned comparison revealed that in the first two minutes, older participants tended to generate more solutions related to expansion (*M* = 1.40, *SD* = 1.03) than did young adults did (*M* = 1.08, *SD* = 1,04, *t*(242) = 1.83, *p* =.07, *d* = 0.31) at T1. In contrast, the older adults proposed fewer solutions related to fixation (*M* = 2.34, *SD* = 1.28) than did the younger adults (*M* = 2.87, *SD* = 1.40, *t*(242) = 2.42, *p* =.02, *d* = 0.39) and middle-aged adults (*M* = 3.04, *SD* = 1.44, *t*(242) = 3.21, *p* =.002, *d* = 0.51) at T1. Finally, in the last two minutes (T5), middle-aged adults generated more ideas related to fixation (*M* = 0.53, *SD* = 0.93) than did older adults (*M* = 0.27, *SD* = 0.62, *t*(242) = 2.37, *p* =.02, *d* = 0.32) and young adults (*M* = 0.18, *SD* = 0.39, *t*(242) = 3.28, *p* =.001, *d* = 0.48). The other comparisons between age groups were not significant (all p values > 0.05) Fig 2.

To determine whether the ideas generated by older adults also had higher originality scores, we performed a one-way ANOVA on the originality score. The results of the ANOVA revealed a main effect of age group (*F*(2, 242) = 3.16; *p* =.04, *η²p* =.03). The post hoc analysis revealed that older adults (*M* = 2.46; *SD* = 0.70) had higher originality scores than younger adults did (*M* = 2.21; *SD* = 0.72, *t*(242) = 2.27, *p* =.02, *d* = 0.35) and middle-aged adults did (*M* = 2.23; *SD* = 0.67, *t*(242) = 2.11, *p* =.04, *d* = 0.33). There was no significant difference found between middle-aged adults and younger adults (*p* =.86).

**Fig. 3:**
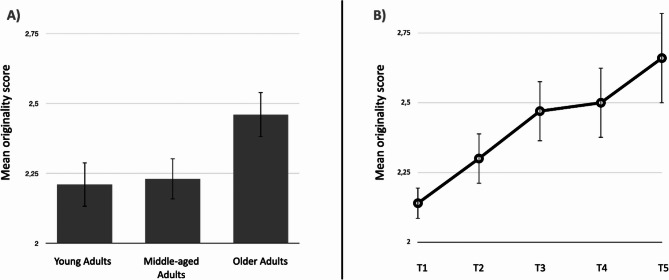
The mean originality of responses across age group (**A**) and over time (**B**)

As many participants did not provide both fixation and expansion responses toward the end of the task, we were unable to conduct a time-based originality analysis that includes type of idea as a factor. However, when including time as a factor in the analysis of originality, results show a main effect of time (*F*(4, 204) = 4.31; *p* =.002, *η²p* =.08) (Fig. 3) but no interaction between age and time (*F*(8, 204) = 0.36; *p* =.98, *η²p* =.01). Our data support the presence of a serial order effect, as originality increases linearly over time (Fig. 3).

To evaluate the developmental effect on the metacognitive score, we conducted an ANOVA with age group serving as the between-subject factor. There was no significant effect of age on metacognitive control found at the response level (*p* =.44), suggesting that participants similarly overestimate the level of creativity of their ideas, as indicated by a positive MCC-R scores for all age groups (Young adults: *M* = 0.22; *SD* = 0.28; Middle-aged adults: *M* = 0.26; *SD* = 0.25; Older adults: *M* = 0.21; *SD* = 0.29).

**Fig. 4:**
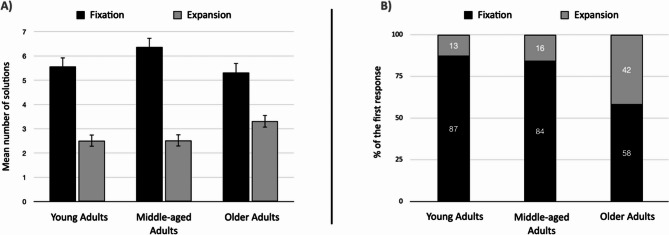
**A** the number of responses in fixation and expansion as a function of age (Error bars represent the standard error of the mean: SEM). **B** the proportion of first response in fixation and expansion as a function of age

To further test H3, we determined whether the very first idea provided was defined as either a fixation or expansion response [14]. We performed a chi-square analysis to compare the effects of age groups on the nature of the first generated idea. The results of the chi-square analysis revealed that the proportion of ideas in expansion and in fixation differed among the three age groups (*χ2* (2) = 21.3, *p* <.001). Both young and middle-aged participants generated more fixation ideas (87% and 84%, respectively), whereas more older people generated more expansion-related first ideas (41.6%) (Fig. 4).

## Discussion

The aim of the present study was to investigate age-related differences in creative problem solving, specifically the ability to resist fixation effects and generate original solutions over time. In contrast with H1, our findings suggest that older adults can surpass younger adults in specific aspects of creative thinking. Compared with both younger adults and middle-aged adults, older adults achieved higher originality scores and expansivity scores. Although these results challenge the traditional view that creativity decreases with age [2–4], they align with H3 and previous research suggesting that older individuals may use more remote associations, compensating for potential declines in executive function with a rich network of prior knowledge [1, 26].

The temporal analysis of idea generation as either fixation and expansion responses provides further insights into the nature of the processes underlying this effect. Compared with younger and middle-aged adults, older adults presented a greater proportion of initial responses related to expansion rather than to fixation. In addition, the analysis demonstrated that older adults provided fewer fixation responses and more expansion responses at the beginning of the task (H3) but not afterward (H2). Older adults’ early non-fixated responses suggest that this advantage might stem from broader and more remote associations in memory rather than from a better capacity to control fixation itself [14]. However, as we did not directly assess semantic memory or associative structure, this interpretation should be regarded as a hypothesis to be tested in future research, for instance using semantic network approaches [35]. In line with this result, but in contrast with previous studies [19], the creative advantage of older adults did not seem to be related to their executive level or their metacognitive monitoring skills, given that the results remained significant even after correcting for WM and I scores [29, 36, 37]. This interpretation is further supported by previous research on the serial order effect [17–19] which shows that executive demand is greater at the end of the task than at the beginning, thereby contrasting with the greater performance observed in older adults at the start of the task. Interestingly, our findings on originality scores revealed a serial order effect in each age group, where later ideas were more creative than earlier ones [17]. However, this increase in originality was found to be due mainly to a decrease in the number of uncreative, fixated solutions rather than an increase in expansive solutions over time. Taken together, these findings also align with recent developments in the triadic model of creativity [14] and suggest that some individuals—due to the associative structure of their knowledge—may be able to intuitively generate expansive responses without the need to inhibit fixation. Supporting this view, a recent study using the two-response paradigm [14], originally developed in the field of reasoning, showed that some participants (both novices and experts) demonstrated a form of creative intuition: they provided spontaneous, unbiased, and creative responses even under time pressure, which is typically assumed to limit access to System 2 processes. In this context, one may speculate that older adults possess greater creative intuition than younger individuals. It would be valuable to directly test this hypothesis in future research using a two-response paradigm, which could help distinguish between spontaneous and deliberative contributions to creative thinking. In addition, Our findings align with the SOC (Selection, Optimization, and Compensation) model [38, 39]. In the context of creativity, the SOC model suggests that older adults may compensate for declines in certain cognitive functions by strategically selecting and optimizing resources, allowing them to maintain or even enhance performance in specific domains. This perspective helps to explain why some older individuals may continue to produce highly original ideas, potentially by relying on accumulated knowledge and adaptive strategies that support creative thinking despite age-related changes in cognitive control.

However, our research is not without limitations. First, this study was conducted fully online, which prevented us from obtaining more conventional measures of executive function. Even though older adults did not show deficits in executive function assessment questionnaires, it will be essential to replicate these findings using more conventional experimental measures, such as the Stroop or stop-signal tasks. Such exploration is particularly important given that some research suggests that distant associative abilities may stem from reduced executive control. While our data do not support this interpretation, we cannot exclude this possibility without examining participants’ executive performance using objective measures. The absence of an age effect on metacognitive control may also suggest that the ability to detect original ideas does not rely on deliberative processes. This interpretation is consistent with findings from the reasoning literature [40], which have shown that conflict detection is an intuitive process. Further studies are needed to confirm this in the domain of creativity, for instance by using a dual-task paradigm during participants’ evaluation of creativity.

Future studies might also benefit from examining how specific executive functions interact with associative processes to support creativity throughout adulthood. In this vein, it would be necessary to evaluate the structure of participants’ semantic networks [35], specifically those related to the problem they are tasked with solving, to confirm the interpretative hypothesis of a superior capacity for distant associations in older adults. Although the fact that older adults are able to provide responses beyond fixation from the very first minutes suggests that their performance is not merely the result of cognitive fatigue, further research is needed to clarify the role of executive resources. Such studies would help determine whether specific features of associative memory may compensate for age-related declines in executive functioning. Furthermore, we did not collect information on the participants’ physical and mental health, which may also impact their creativity and executive functions. Moreover, the egg task has specific features that distinguish it from the divergent thinking measures typically used in previous studies. Indeed, the egg task requires participants to diverge in order to solve a specific problem, rather than simply to be creative. As such, it may place greater demands on abstract reasoning compared to classical divergent thinking tasks. However, a recent study suggests that older adults may exhibit greater originality than younger individuals even when using the Alternative Uses Task (AUT) [41]. Finally, given the online status of the study, it is possible that the older adults who consented to do this study had more experience with digital tools than the rest of the population, which may reduce the generalizability of the findings. Thus, further studies are needed to replicate these findings in more traditional in-person laboratory settings or to account for participants’ digital literacy.

## Conclusion

The present study highlights that older adults may outperform younger adults in certain aspects of creative problem solving, specifically in regard to generating original and expansive responses early in the task. This advantage appears to be more linked to broader and more remote associations in memory than to executive function. These findings have practical implications for both management and education. They suggest that older adults, due to their lower initial fixation, may be valuable assets in teams aiming to develop creative projects. It also appears crucial to consider age more carefully when designing cognitive tools intended to foster idea generation. While younger individuals may benefit from strengthening their ability to resist initial intuitive responses, older adults might be better supported by helping them build upon their creative intuition. Beyond these practical insights, our results can also be interpreted within the framework of the SOC model [38, 39], which provides a useful perspective on how to leverage age-related strengths in creative contexts. From this viewpoint, mixed-age teams could benefit from structures that allow older members to select idea domains aligned with their expertise and associative richness, optimize idea generation through feedback and intergenerational knowledge exchange, and compensate for potential declines in executive control by using external aids or complementary team roles. Such SOC-based strategies could help balance cognitive diversity and sustain creative productivity, allowing older and younger members to contribute distinct yet synergistic strengths to the creative process. Taken together, these results confirm the need to move beyond certain stereotypes that we hold about older adults and offer new research directions with respect to the involvement of both associative and executive processes in creative problem solving.

## Data Availability

The datasets analyzed during the current study are available at: https://osf.io/a4d9n/files/osfstorage/684ab719c2209fbf4b85b35a.
